# Pigmented Basal Cell Carcinoma of Nipple and Areola in a Male Breast - A Case Report with Review of Literature

**Published:** 2014-03

**Authors:** R. Kalyani, B. R. Vani, Murthy V. Srinivas, P. Veda

**Affiliations:** ESIC Medical College & PGIMSR, Bangalore, India

**Keywords:** Basal cell carcinoma-nipple and areola complex, male breast, skin cancer

## Abstract

Basal cell carcinoma is a common skin cancer worldwide. However basal cell carcinoma of nipple and areola complex is rare, commonly seen in males in elderly age group. The tumor has aggressive behavior with increased tendency for metastasis. We present a case in a 78 year male in the left breast.

## INTRODUCTION

Skin cancers are the most common cancers accounting for half of all human cancers. Of these, non-melanoma skin cancers are common and basal cell carcinoma (BCC) accounts for 70-80% of all cases. Basal cell carcinoma of nipple and areola complex (BCC-NAC) is a very rare site (1-5). The incidence of BCC-NAC has doubled in past one decade which is thought to be because of increased awareness and detection of skin cancer (1). The median age of BCC-NAC is elderly age about 60 years as in other BCC (4). The cause for BCC is multifactorial of which exposure to ultraviolet radiation in sun exposed areas as head and neck is common especially in light skinned people. However BCC-NAC occurs in sum protected areas (1-6). BCC-NAC is deceptive in clinical presentation / aggressive behavior and can persist for long duration as much as 10 years (4). We present a case of Pigmented BCC-NAC, in the left breast in a 78 year male.

## CASE REPORT

A 78 year old male, RAN, from Bangalore, India, presented with a pigmented lesion over left chest wall associated with itching and burning sensation since 4 months, gradually increasing in size. Local examination showed pigmented macular lesion involving nipple and areola of left breast. No lymphadenopathy detected. No similar lesion found elsewhere in the body. No past history of exposure to radiation, arsenic or tar. No past history of burns, surgery, trauma, disease of nipple or skin cancer. No family history of BCC or skin cancer. Patient blood group was B positive. A provisional clinical diagnosis of melanoma was made. Mammography and ultrasound findings were not contributory.

**Figure 1:**
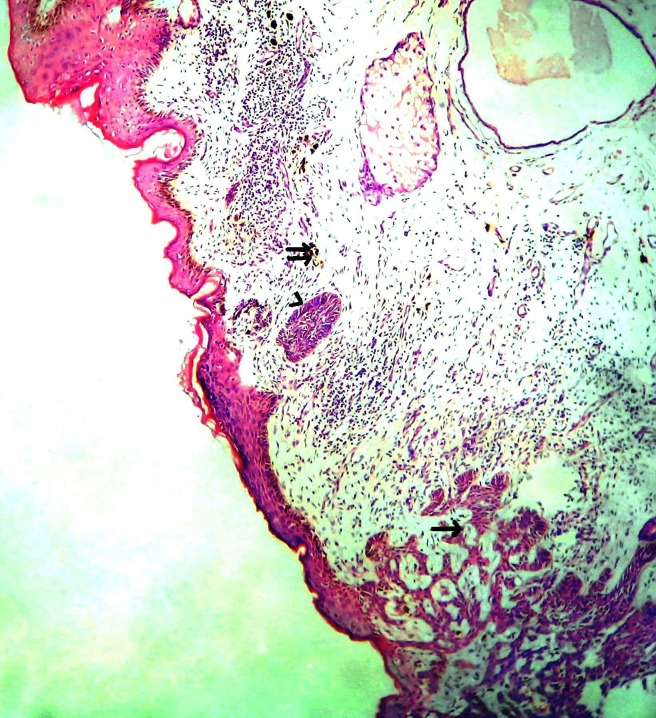
Microphotograph showing epidermis and dermis, lower end shows tumour cells in complex branching cords (single arrow) with upper end showing melanophages (double arrow) and focal tumour cells showing pallisading pattern (arrow head). H&E × 50.

**Figure 2:**
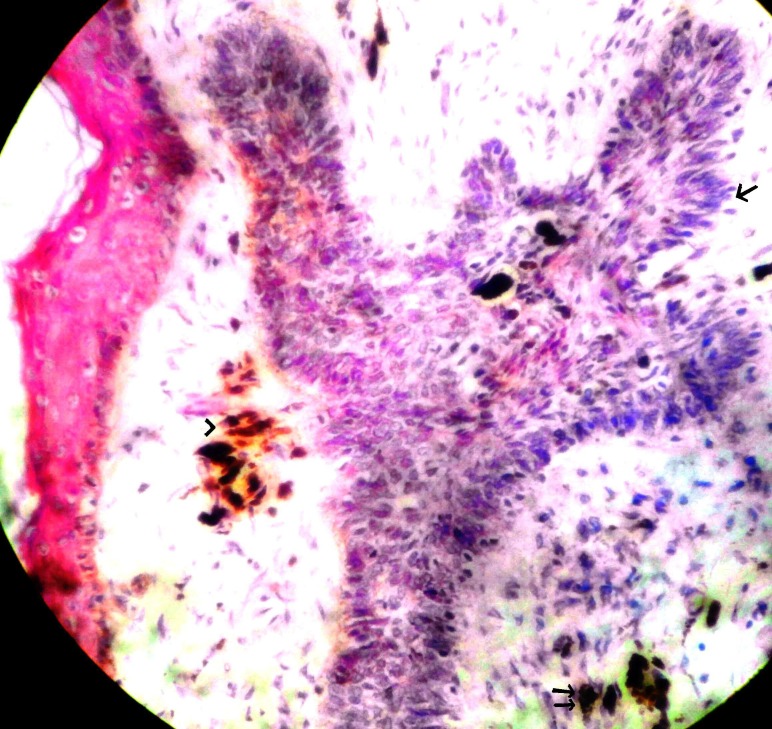
Microphotograph showing tumour cells in cords with peripheral pallisading pattern (single arrow) and melanin pigment in the tumour cells (double arrow) and in stroma (arrow head). H&E × 100.

**Figure 3:**
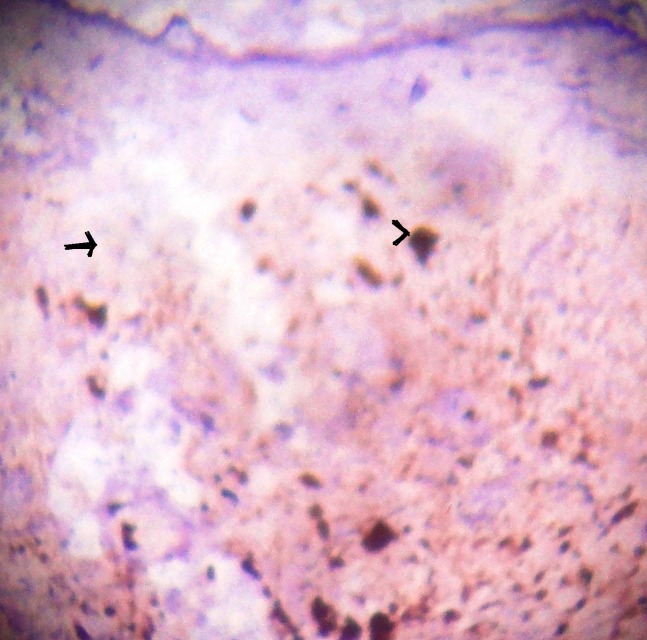
Microphotograph of tissue section with HMB 45 marker which shows negative in tumour cells (arrow) and positive with melanin pigment (arrow head). HMB45 × 100.

**Figure 4:**
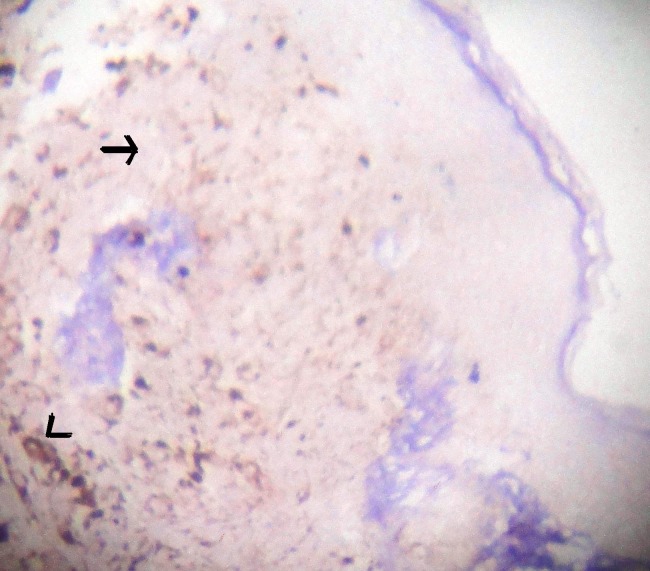
Microphotograph of tissue section with S100 marker which shows negative in tumour cells (arrow) and positive with melanin pigment (arrow head). S100 × 100.

Patient underwent surgical three dimensional excision along with nipple and areola with one cm margin and primary closure. The specimen was subjected to histopathological examination. Gross specimen consisted of elliptical skin with subcutis measuring 7 × 3.5 × 2 cms with nipple and areola. Pigmented lesion over nipple and areola measured 1 × 0.5 cms and showed ulceration. Cut section over pigmented lesion showed grey white to grey black areas. The tissue bits were processed by routine processing method using increasing grades isopropyl alcohol as dehydrating agent, xylene as clearing agent, embedded in paraffin wax and tissue blocks were prepared with paraffin wax. Tissue sections were taken from paraffin blocks, which were dewaxed by xylene, dehydrated with isopropyl alcohol and stained with hematoxylin and eosin stain by routine conventional method. Microscopy showed epidermis and dermis. Epidermis showed proliferating basaloid cells, extending into dermis. The tumour cells were round to oval with vesicular nuclei and scanty cytoplasm with peripheral pallisading arrangement. The tumour cells were also arranged as interconnecting cords in between myxoid matrix. Focal areas showed melanin pigment in tumour cells. Stroma showed infiltration by chronic inflammatory cells and melanophages (Fig. 1 and Fig. 2). Foreign body giant cell reaction was seen. The resected surgical margins were free from the tumour. Immunohistochemistry of tissue sections were negative for HMB45 (Fig. 3) and S100 (Fig. 4) and positive for pankeratin. A final histopathological diagnosis of Pigmented Basal cell carcinoma of nipple and areola was made. Patient was followed up for one year, no recurrence or metastasis noted.

## DISCUSSION

BCC is the common malignancy of skin especially in western world and the incidence continues to increase worldwide (1-3, 5). Basal cell carcinoma of nipple areola complex (BCC-NAC) is extremely rare and increasing (1-5, 7). Robinson reported first case of a rodent ulcer of the male breast in a 60 year old male in 1893 (3, 8). From 1983 to 2011 40 cases are reported in English literature; of which 25 cases are males with one third of cases were solely with nipple involvement. (1, 7).

BCC-NAC in males is commonly seen between 43-80 years of age and in females between 49-75 years of age. The male: Female ratio is 1:1 in BCC for common sites but BCC-NAC shows male preponderance which is thought to be because of increased exposure of chest area to sun light (1, 3-7). BCC-NAC is more common on left breast than right (5). BCC is common in sun exposed areas as head and neck. The sites of BCC in covered areas or low exposure to ultraviolet ray exposure are medial quadrant of orbit, axila, groin, palm, thigh, feet, genital and perineal region which are rare sites (2, 3). BCC-NAC is a very rare site (3). The other predisposing factors are immunocompromised status, exposure to radiation, arsenic, tar, prior burns, genetic predisposition, sunbathing, previous history of surgery, trauma, pre-existing disease of the nipple, BCC in other sites, family history of BCC/skin cancer, basal cell nevus (Gorlin’s) syndrome, tendency to sun burn, aging, x-ray exposure and history of acinic keratosis (1-3, 5-7). Many times BCC-NAC arises as de-novo lesion (2). In the present case the patient is a 78 year male with the lesion in left breast. History did not reveal any risk factors except for age factor.

The BCC-NAC present as erythematous lesion, scaling, ulceration, eczema, subareolar mass, plaques, papules, eczematous lesion, nodular mass or crusty ulcer. Sometimes associated with lymphadenopathy (1, 2, 6). Histologically BCC-NAC shows proliferating nests of basaloid cells arising from the epidermis and extends into superficial dermis and nipple stroma (Fig. 1). Often tumour nests involve underlying lactiferous ducts. Pertitumour cleft seen between tumour nests and stroma. Sometimes melanin pigment seen in tumour cells and stromal macrophages (Fig. 2) (4, 5, 7). BCC-NAC has no predilection of a particular type of BCC. Majority of the reported cases are of superficial type followed by nodular or solid type. Pigmented type is reported in 3 cases. Other types reported are ulcerated, kerototic infiltrative, fibroepithelioma and multicenteric type. Superficial BCC is not an aggressive subtype but difficult to eradicate because of presence of many plates of tumour cells attached to basal epidermis, multifocal, interconnected in 3 dimensions. Prognosis is good by excision and occasionally supplemented by irradiation. Infiltrative subtype has an increased potential for recurrence/ metastasis (6, 7). In the present case the patient presented as pigmented and ulcerated lesion involving nipple and areola. Histopathology showed classical features of basal cell carcinoma with melanin pigment within and outside tumour cells.

Ultrasound in BCC-NAC usually does not show any mass. Mammography may show microcalcification in periareaolar area or in the nipple with features of involution of breast tissue. However quite often no findings made out in mammography (1, 5, 7). Ultrasound and mammography was not contributory in the present case.

The differential diagnosis of BCC-NAC are Paget’s disease, Bowmen’s disease, erosive adenomatosis, contact/chronic dermatitis, eczema, adenoma of nipple, papilloma of the lactiferous duct, syringomatous adenoma, invasive ductal carcinoma, malignant melanoma and other malignant skin tumour (1, 3-5, 7). In the present case provisional clinical diagnosis was melanoma. HMB45 and S100 markers were done to completely rule out melanoma.

BCC is locally invasive and rarely metastasize (0.0028 to 0.5%) However BCC-NAC has increased metastatic potential of 9.1 to 11.5% and highly aggressive (1, 2, 7). The increased metastatic potential is due to rich angiolymphatic vessels in subareaolar region which give rise to easy tumour spread to lymph nodes. Hence sentinel lymph node margination surgery to be considered along with the surgical treatment (1, 3, 4, 7). Another explanation for increased metastatic potential in BCC-NAC is due to increased prosperity of the tumour to invade underlying lactiferous ducts and hence potentially invade the deep soft tissue unlike BCC of other areas giving rise to increased chance of metastasis (3, 4, 7). Hence special attention should be given in treatment of BCC-NAC (1, 7). Large and ulcerated BCC-NAC has showed increased metastasis (1). Hence long period of follow-up is advisable in BCC-NAC for recurrence and metastasis (4, 7). However, based on reported cases and follow-up for 8 years, one study suggests that BCC-NAC does not have different or greater malignant potential than BCC of other sites (6). Treatment of BCC-NAC depends on extent of the tumor and involvement of anatomical structures i.e. the lymph nodes and deeper soft tissue (3, 6). Treatment ranges from medical treatment, radiotherapy, laser, wide excision, simple mastectomy with lymph node dissection. In the present case no lymphadenopathy / involvement of lactiferous duct was noted. The resected margins were free from the tumour. Patient underwent surgical excision with one cm margin and was followed for one year which was uneventful. Treatment and prognosis in various case reports is shown in Table 1.

**Table 1:** Shows the treatment and prognosis in different case reports

Sl. No	Case reports	Treatment	Prognosis

1.	Yasemin Oram, *et al*. 2011	Patient refused large excision.	Patient lost for follow-up.
2.	Abhishek Sharma, *et al*. 2011	Excision biopsy.	No follow-up mentioned.
3.	Ching-Wen Hung, *et al*. 2005	Partial mastectomy with sentinel lymph node excision.	No recurrence after one year of follow-up.
4.	Chakshu Gupta, *et al*. 2004	Excision biopsy done.	Follow-up not mentioned.
5.	Hitoshi Yamamoto, *et al*. 2001	Resection with 2 cms free margin.	No recurrence after 2 years of surgery.
6.	Roberto Betti, *et al*. 2003 (Three cases)	a. Excision of mass. b. Excision of tumour with nipple and areola. c. Excision of tumour with underlying mammary tissue.	a. No recurrence after 5 years. b. No recurrence after 2 years. c. No recurrence after 2 years.
7.	Han Jin Jung, *et al*. 2011	Wide excision with 5 mm safe margin.	No recurrence after 4 months of follow-up.
8.	Present case	Three dimension excision with nipple and areola and one cm safe margin.	No recurrence noted after 1 year follow-up.

To conclude, BCC-NAC is a rare tumour. One should be aware of occurrence of BCC in this unexposed region and also be able to differentiate it from other benign and malignant tumours common in this region by clinical features, histopathological examination and immunohistochemical methods. This case is presented for its rarity and as far as our knowledge goes in the English literature this is the fourth case of pigmented BCC-NAC.
